# Aggregatibacter aphrophilus Septic Arthritis of the Knee With Negative Initial Cultures: A Case Report

**DOI:** 10.7759/cureus.118017

**Published:** 2026-10-16

**Authors:** Sola Maria Saleh, Tanvi Manohar, Ramez Azzam, Suhel Sabunwala

**Affiliations:** 1 Department of Infectious Diseases, University of Florida, Gainesville, USA; 2 Department of Infectious Diseases, University of Florida College of Medicine, Gainesville, USA

**Keywords:** aggregatibacter aphrophilus, antibiotics, arthrocentesis, infection, septic arthritis

## Abstract

*Aggregatibacter aphrophilus* is a fastidious Gram-negative coccobacillus of the HACEK group that colonizes the oral cavity and is best recognized as a cause of infective endocarditis. Septic arthritis caused by this organism is uncommon, particularly when involving the native knee. We report a 60-year-old man who presented with two weeks of progressive right knee pain and swelling. He was afebrile with a normal peripheral white blood cell count, although inflammatory markers were elevated. Synovial fluid analysis demonstrated 81,000 nucleated cells/µL with 88% polymorphonuclear leukocytes, but Gram stain and initial synovial fluid culture were negative. Arthrotomy with anterior synovectomy and drainage revealed approximately 50 mL of purulent fluid. Operative culture subsequently grew *A. aphrophilus* after five days of incubation, identified by matrix-assisted laser desorption/ionization time-of-flight mass spectrometry. Blood cultures remained negative, and transthoracic echocardiography showed no evidence of valvular vegetation. Dental evaluation demonstrated periodontal disease, although no definitive odontogenic source was identified. The patient was treated with surgical drainage followed by four weeks of intravenous ceftriaxone, with complete clinical recovery. This case highlights the diagnostic challenge of *A. aphrophilus* septic arthritis and the importance of operative cultures when clinical suspicion remains high despite negative initial microbiologic studies.

## Introduction

*Aggregatibacter aphrophilus* is a fastidious Gram-negative coccobacillus that colonizes the human oral cavity and belongs to the HACEK group of organisms [1]. Previously classified as *Haemophilus aphrophilus*, with *Haemophilus paraphrophilus* subsequently incorporated into the same species, *A. aphrophilus* is best recognized for its association with infective endocarditis [1]. However, invasive infections outside the cardiovascular system have also been described, including osteomyelitis, discitis, abscesses, and septic arthritis [2,3]. In a review of 28 invasive *H. aphrophilus* infections, bone and joint infections accounted for approximately one-third of cases, although this group included several forms of osteoarticular infection rather than septic arthritis alone [2].

Native-joint septic arthritis caused by *A. aphrophilus* remains uncommon, with native-knee involvement reported only infrequently in the literature [4-6]. The organism presents an additional diagnostic challenge because of its fastidious growth characteristics. Initial Gram stain and cultures may therefore be unrevealing, potentially delaying microbiologic identification despite clinical and synovial fluid findings consistent with infection [5,6]. This is particularly relevant in septic arthritis, where timely recognition, appropriate microbiologic sampling, antimicrobial therapy, and source control are important to prevent joint destruction and other complications [7].

The oral cavity represents a potential source of invasive *A. aphrophilus* infection. Dental disease and recent dental procedures have been reported in patients with invasive infection, supporting hematogenous dissemination as a possible mechanism [2,3]. Because *A. aphrophilus* is also a recognized cause of infective endocarditis, its recovery from a normally sterile site warrants consideration of an endovascular source when supported by the clinical presentation.

We report a case of *A. aphrophilus* native-knee septic arthritis in a 60-year-old man who presented with progressive knee pain and swelling despite being afebrile and having a normal peripheral white blood cell count. Initial synovial fluid Gram stain and culture and blood cultures were negative, while operative exploration revealed a substantial purulent collection and ultimately provided the specimen from which *A. aphrophilus* was identified. The case illustrates the diagnostic challenges associated with this fastidious organism and the importance of obtaining adequate operative specimens when septic arthritis remains strongly suspected despite initially negative microbiologic studies.

## Case presentation

A 60-year-old male with a medical history of ulcerative colitis treated with mesalamine, proctitis, and prostate cancer in remission, presented with complaints of right knee pain and swelling. His symptoms began approximately two weeks before presentation and acutely worsened two days before admission. He denied preceding trauma, falls, or injury. Approximately 30 years earlier, he had undergone anterior cruciate ligament (ACL) reconstruction of the same knee using a patellar bone-tendon autograft, without subsequent complications. At presentation, the patient was afebrile (98.1°F), with a blood pressure of 134/77 mm Hg, heart rate of 104 beats/min, respiratory rate of 14 breaths/min, and oxygen saturation of 95% on room air. He denied recent fever, chills, nausea, vomiting, night sweats, abdominal pain, diarrhea, bloody stools, hospitalization, or illness. He was not sexually active. He worked as a general contractor, which required frequent ladder climbing and heavy lifting.

On examination, the skin over the right knee was intact, with mild-to-moderate swelling compared with the contralateral knee. Surgical incisions from the prior ACL reconstruction were well healed, without erythema, dehiscence, or other wound abnormalities. Sensation was intact in the superficial peroneal, deep peroneal, sural, saphenous, and tibial nerve distributions. The dorsalis pedis pulse was 2+, and the compartments of the right lower extremity were soft and compressible. The patient had a near-full passive range of motion without significant pain, although mild pain occurred with terminal extension. He was able to bear weight with minimal discomfort. Initial laboratory findings are summarized in Table 1.

**Table 1:** Selected laboratory findings at presentation

Labs	Value	Normal Range
White Blood Cell count	7.7 x 10^3^/ microliter	4–10 x 10^3^/ microliter
RBC (Red Blood Cell count)	4.95 x 10^6^/ microliter	4.50–5.90 x 10^6^/ microliter
Hemoglobin	13.9 grams/ deciliter	13–16.5 grams/ deciliter
Platelet Count	267 x 10^3^/ microliter	150–450 x 10^3^/ microliter
Creatinine	1.36 milligrams/deciliter	0.51–1.18 milligrams/deciliter
Erythrocyte Sedimentation Rate	97 millimeters per hour	0–10 millimeters per hour

Radiography of the right knee demonstrated postsurgical changes from prior ACL reconstruction, moderate joint effusion, and overlying soft-tissue edema (Figure 1).

**Figure 1:**
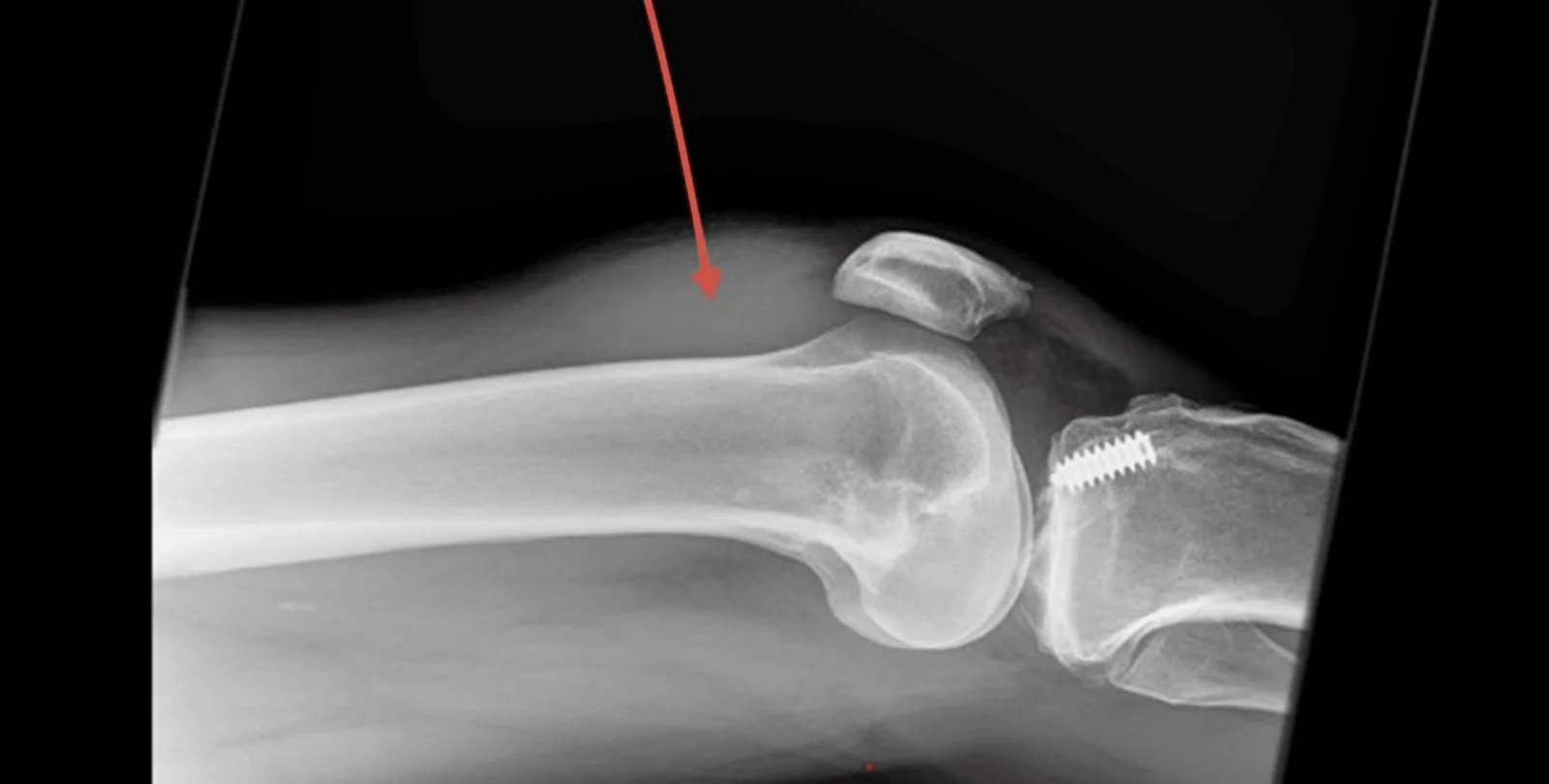
X-ray of the right knee showing joint effusion The image shows no evidence of fracture or traumatic subluxation. It shows post-surgical changes of prior anterior cruciate ligament (ACL) reconstruction. There is moderate joint effusion (marked with a red arrow) with overlying soft tissue edema.

Computed tomography (CT) showed no acute osseous abnormality and demonstrated postsurgical changes, osteoarthritic changes, and a small joint effusion (Figures 2, 3).

**Figure 2:**
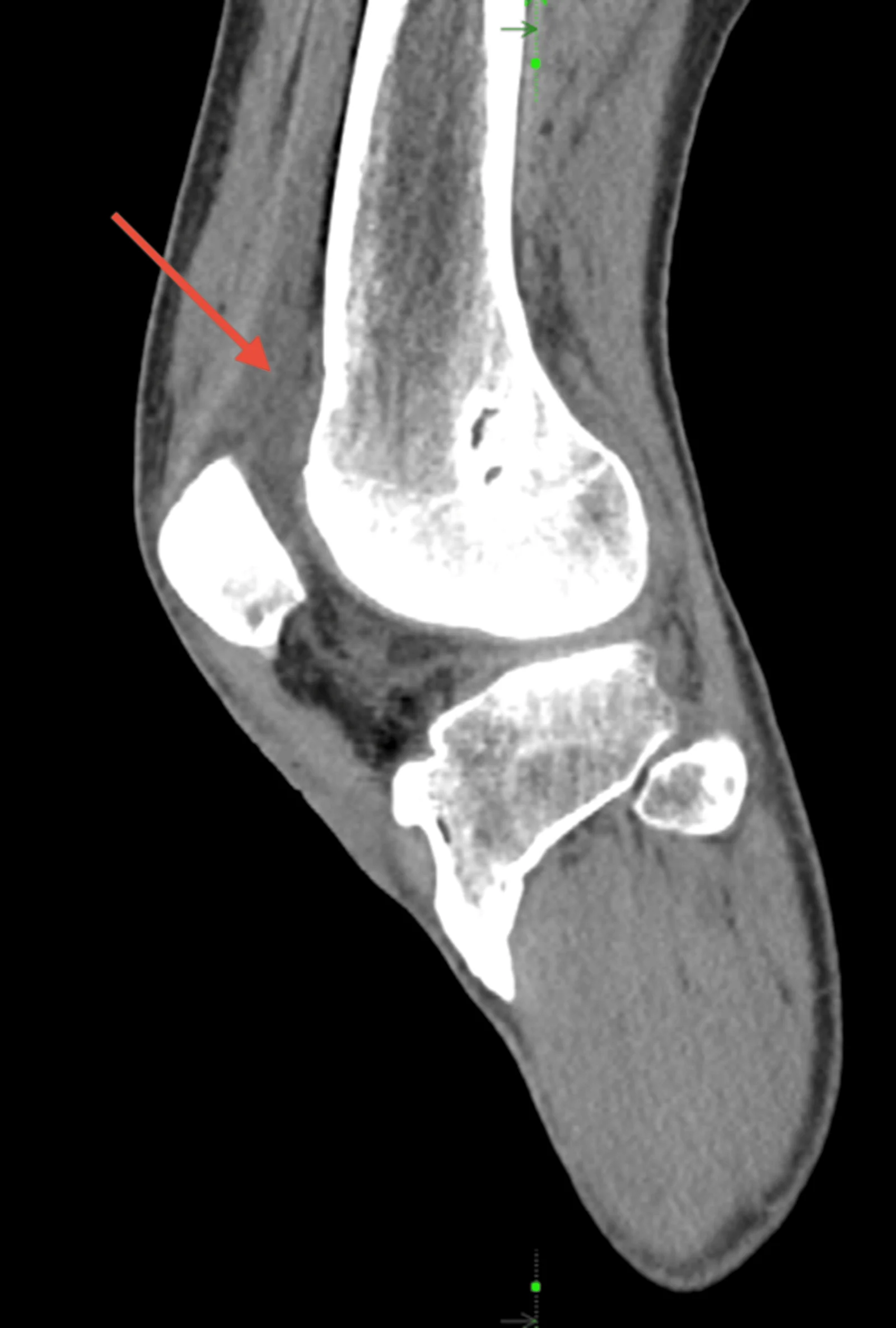
CT scan sagittal section of the right knee

**Figure 3:**
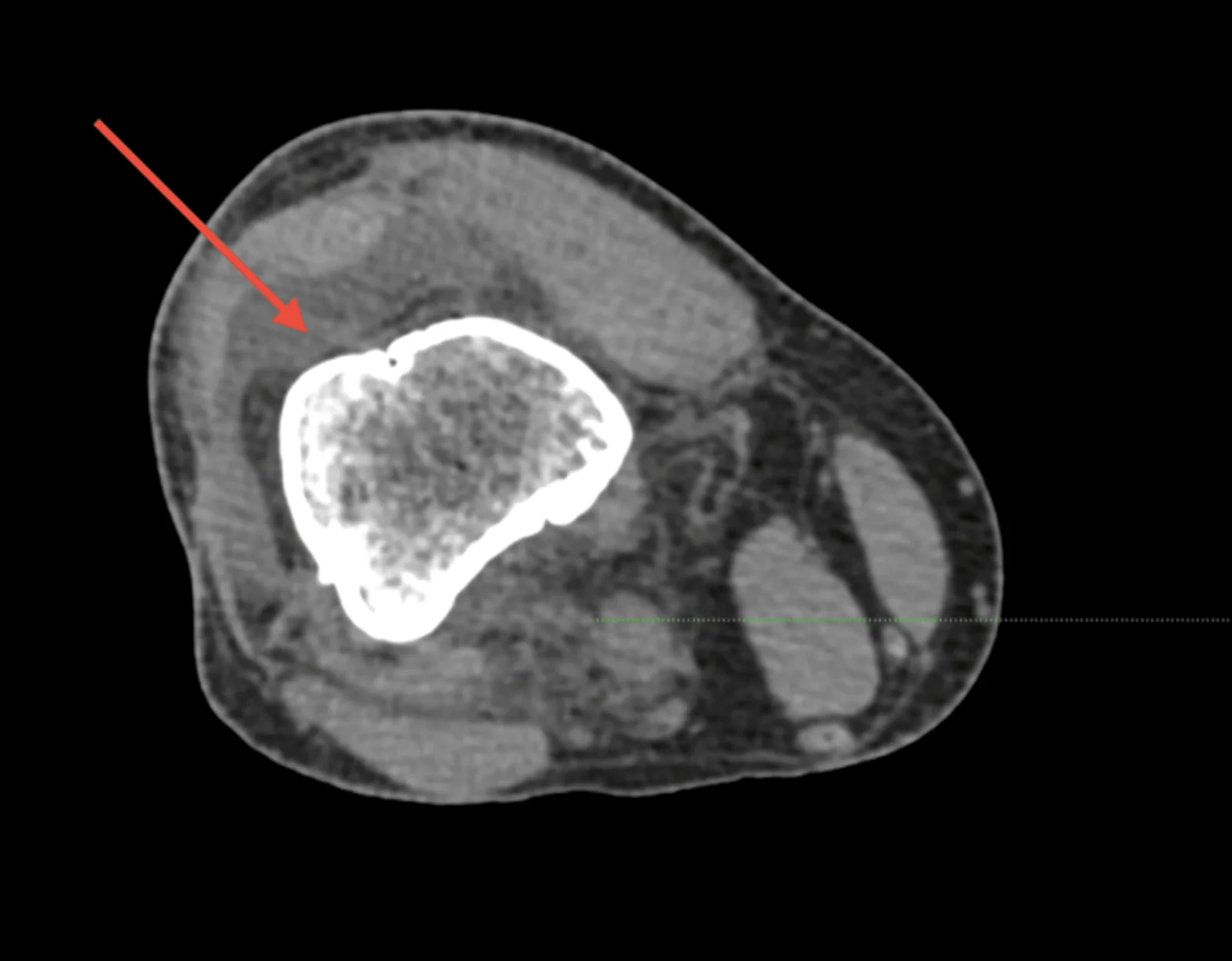
CT scan transverse section of the right knee CT image shows no acute osseous abnormalities. Postsurgical changes of anterior cruciate ligament reconstruction, trace tricompartment osteoarthritis, and a small joint effusion are present (marked with a red arrow).

Arthrocentesis of the right knee was performed using a 19-gauge needle, yielding 12 mL of bloody, serous-appearing fluid (Figure 4).

**Figure 4:**
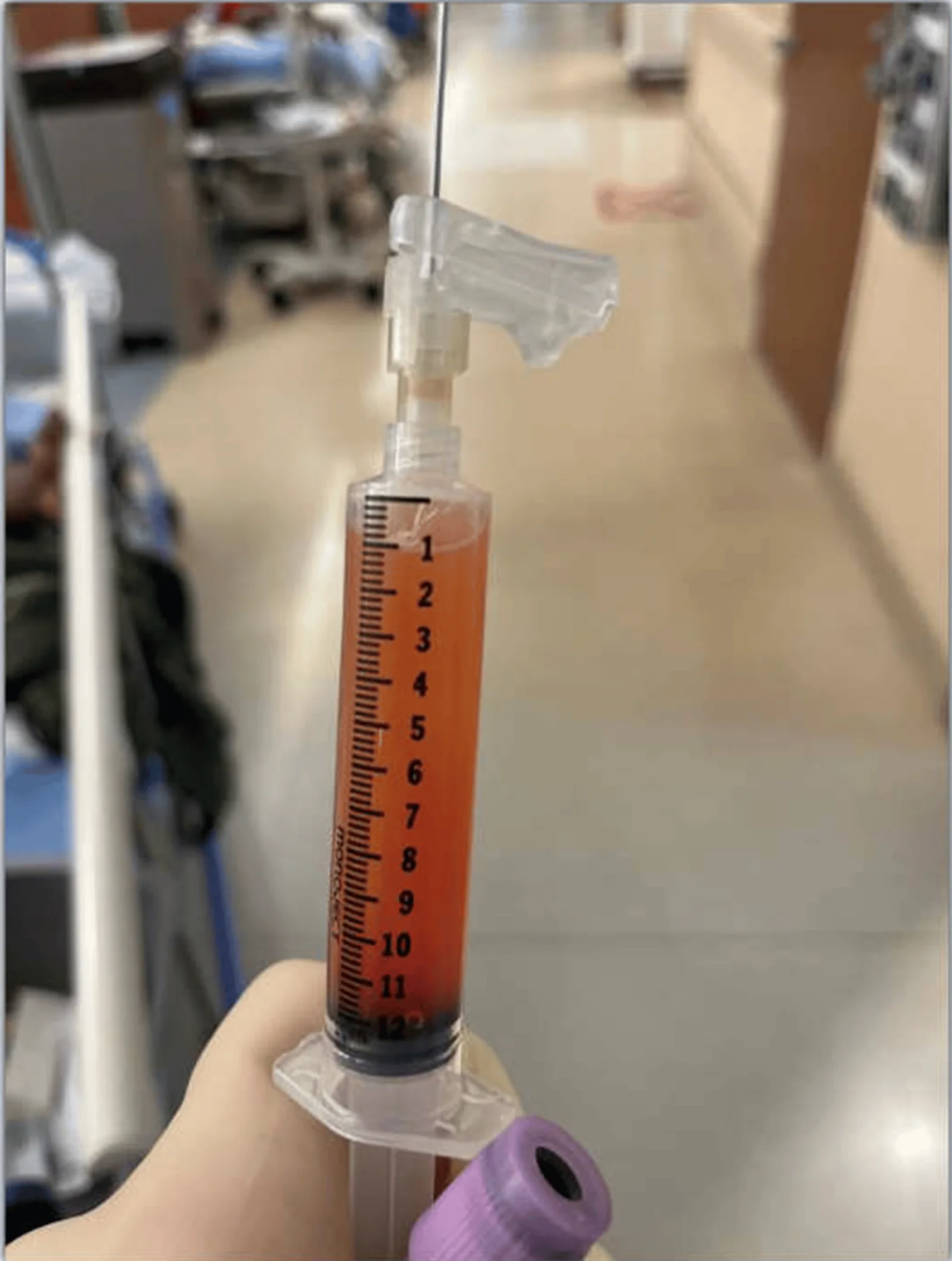
Synovial fluid obtained during right knee arthrocentesis

Synovial fluid was sent for cell count, Gram stain, culture, and crystal analysis. The synovial fluid contained 81,000 nucleated cells/µL, with 88% polymorphonuclear leukocytes. No crystals or organisms were identified, and the initial synovial fluid culture showed no growth (Table 2).

**Table 2:** Synovial fluid and microbiologic findings MALDI-TOF MS: matrix-assisted laser desorption/ionization time-of-flight mass spectrometry

Finding	Result
Synovial nucleated cell count	81,000 cells/µL
Polymorphonuclear leukocytes	88%
Crystals	None identified
Initial synovial fluid Gram stain	White blood cells present; no organisms identified
Initial synovial fluid culture	No growth
Operative specimen Gram stain	No organisms identified
Operative specimen culture	*Aggregatibacter aphrophilus*, identified after five days of incubation by MALDI-TOF MS
Blood cultures	No growth

Given the concern for septic arthritis, orthopedic surgery was consulted. The patient underwent right knee arthrotomy with anterior synovectomy and drainage of a deep abscess. Approximately 50 mL of purulent synovial fluid was encountered intraoperatively. The joint was irrigated and debrided with 5 L of normal saline. Gram staining of the operative specimen showed no organisms.

Following surgery, empiric intravenous vancomycin and cefepime were initiated while culture results were pending. His knee pain improved postoperatively. After five days of incubation, small, rough colonies grew from the operative specimen and were identified as *Aggregatibacter aphrophilus* using matrix-assisted laser desorption/ionization time-of-flight mass spectrometry (MALDI-TOF MS).

Antimicrobial therapy was subsequently narrowed to ceftriaxone 2 g intravenously once daily. Blood cultures obtained on admission before antibiotic administration remained negative. Given the association of *A. aphrophilus* with infective endocarditis, transthoracic echocardiography was performed and showed no valvular vegetations.

The patient reported undergoing a dental cleaning approximately one month before presentation. Oral examination was notable for poor oral hygiene and a fractured porcelain crown of tooth #31 (right mandibular second molar). Panoramic dental imaging demonstrated generalized periodontal bone loss, periapical rarefying osteitis at tooth #23, and possible caries. He was subsequently evaluated by oral and maxillofacial surgery, with no acute odontogenic infection identified that required intervention. Although a definitive source could not be established, transient bacteremia from the oral cavity was considered a possible mechanism of hematogenous seeding, and he was discharged with a PICC line (peripherally inserted central catheter) with plans to continue IV Ceftriaxone for four weeks.

The patient was discharged with a peripherally inserted central catheter (PICC) to complete four weeks of intravenous ceftriaxone. At follow-up four weeks after discharge, he had completed antimicrobial therapy, and the PICC was removed. He reported no knee pain, fever, wound drainage, or swelling. He was able to bear weight and ambulate independently without an assistive device.

## Discussion

Native knee septic arthritis caused by *A. aphrophilus* is particularly uncommon. Previous reports span several decades and include infections described under both the current name and its former taxonomic classifications (Table 3) [4-6].

**Table 3:** Reported native-knee septic arthritis caused by Aggregatibacter aphrophilus and its former taxonomic classifications A. aphrophilus: Aggregatibacter aphrophilus; CRP: C-reactive protein; IV: intravenous.

Report	Age/Sex	Joint	Organism reported	Microbiologic findings	Treatment	Outcome / notable feature
von Essen et al., 1987 [6]	Adolescent male	Knee	Haemophilus paraphrophilus	Organism recovered using an improved culture protocol; historical species later incorporated into A. aphrophilus	Antimicrobial treatment	Recovery reported; highlighted difficulty of microbiologic recovery
Merino et al., 1994 [4]	Middle-aged man	Right knee	Haemophilus aphrophilus	H. aphrophilus isolated as the cause of septic arthritis	Ceftriaxone	Full recovery without sequelae
Prestifilippo et al., 2019 [5]	17-year-old male	Left knee	Aggregatibacter aphrophilus	Blood and initial joint-fluid cultures negative; organism recovered from operative specimens	Arthrotomy and ceftriaxone	Favorable clinical evolution
Present case, 2026	60-year-old man	Right knee	Aggregatibacter aphrophilus	Synovial Gram stain and blood cultures negative; operative tissue culture positive	Arthrotomy, anterior synovectomy and abscess drainage; ceftriaxone 2 g IV daily for 4 weeks	Pain-free weight-bearing; CRP decreased from 121.58 to 2.31

Among the reported peripheral-joint infections, only a small number have involved the native knee. Merino et al. described knee septic arthritis caused by *H. aphrophilus* in a middle-aged man in 1994 [4], while Prestifilippo et al. reported *A. aphrophilus* knee septic arthritis in a 17-year-old male in 2019 [5]. An earlier knee infection reported by von Essen et al. as *H. paraphrophilus* is also relevant because this organism was subsequently incorporated into *A. aphrophilus* [1,6]. As shown in Table 3, our patient differs from the previously reported knee cases in his older age and the operative finding of a substantial purulent collection, while sharing the important finding that bacteremia may be absent.

The microbiologic findings in our patient are particularly relevant. Despite an afebrile presentation and normal peripheral white blood cell count, the patient had markedly elevated inflammatory markers, and a synovial fluid leukocyte count of 81,000 cells/mm³ with 88% neutrophils. No organisms were identified on the initial Gram stain, and blood cultures remained negative. A synovial leukocyte count above 50,000 cells/mm³ is considered suggestive of native-joint septic arthritis, although it is not diagnostic in isolation, and Gram staining has limited sensitivity [8]. At surgery, however, approximately 50 mL of purulent synovial fluid and a deep abscess were encountered, and *A. aphrophilus* was ultimately recovered from operative tissue. A similar diagnostic challenge was described by Prestifilippo et al., whose patient had negative blood and initial joint-fluid cultures before *A. aphrophilus* was recovered from specimens obtained during arthrotomy [5]. The earlier report by von Essen et al. also emphasized the difficulty of recovering the organism and the importance of appropriate culture methods [6]. These cases show that negative initial microbiologic studies should not outweigh a clinical picture strongly suggestive of septic arthritis. Current native-joint septic arthritis guidelines recommend obtaining synovial biopsies for culture when surgical intervention is performed and considering prolonged culture or molecular testing when difficult-to-culture organisms are suspected [8].

The source of infection in our patient could not be established with certainty. *A. aphrophilus* is part of the normal oral flora, and invasive infection has been associated with dental disease and recent dental procedures [2,3]. Huang et al. found that 11 of 28 patients (39%) with invasive *H. aphrophilus* infection reported a recent dental procedure [2]. Bernard et al. also considered the oral cavity the likely source in a patient with lumbar septic arthritis and associated psoas and paravertebral abscesses [3]. In our patient, dental imaging showed periapical rarefying osteitis at tooth #23, possible caries, and generalized periodontal bone loss. However, subsequent evaluation by oral and maxillofacial surgery found no acute odontogenic infection requiring intervention. The oral cavity therefore cannot be established as the source, but transient bacteremia related to underlying periodontal disease remains plausible. Importantly, an oral source does not necessarily require an active dental abscess at the time the invasive infection is recognized.

The patient’s remote anterior cruciate ligament reconstruction approximately 30 years earlier raises a separate question regarding local susceptibility. The long interval between the reconstruction and the current infection argues strongly against a postoperative infection, and there was no evidence that prior orthopedic material represented an active infectious focus. Whether previous reconstruction or chronic structural changes in the knee made the joint more susceptible to hematogenous seeding is uncertain. Given the acute presentation and the known oral reservoir of *A. aphrophilus*, hematogenous spread appears more plausible, although the route of infection cannot be established with certainty.

Isolation of a HACEK organism also raises the possibility of infective endocarditis. In the series reported by Huang et al., seven of 28 patients (25%) with invasive *H. aphrophilus* infection had infective endocarditis [2]. Our patient therefore underwent cardiac evaluation. Blood cultures remained negative, and transthoracic echocardiography showed no apparent vegetation. Echocardiography remains central to the evaluation of suspected infective endocarditis, with transesophageal echocardiography recommended after a negative transthoracic study when clinical suspicion remains high [9]. In our patient, the absence of bacteremia, embolic manifestations, or other clinical findings suggestive of endocarditis, together with improvement after drainage of the knee, made an endovascular source less likely. Nonetheless, the association between *A. aphrophilus* and endocarditis should be kept in mind when the organism is recovered from a normally sterile site, particularly when bacteremia persists, or there are cardiac, vascular, or embolic findings.

There are no organism-specific trials defining the optimal antibiotic regimen or duration for *A. aphrophilus* septic arthritis, and treatment in published cases has been guided largely by antimicrobial susceptibility, the site and extent of infection, and adequacy of source control. Ceftriaxone has been used successfully in reported osteoarticular infections, including cases involving the knee [3-5]. Coburn et al. examined 70 clinical HACEK isolates, including 11 *A. aphrophilus* isolates, and found that all were susceptible to ceftriaxone; susceptibility to some other beta-lactam agents was less consistent [7]. Our patient initially received empiric vancomycin and cefepime, which was narrowed to ceftriaxone after identification of the organism. Arthrotomy, anterior synovectomy, and drainage provided source control, followed by four weeks of ceftriaxone 2 g intravenously every 24 hours. His response was favorable; he returned to pain-free weight-bearing without an assistive device, and CRP decreased from 121.58 at presentation to 2.31 near completion of therapy.

This case adds to the limited literature on *A. aphrophilus* native-knee septic arthritis and illustrates several diagnostic challenges associated with this infection. A substantial purulent collection was present despite an afebrile presentation, normal peripheral white blood cell count, negative blood cultures, and an unrevealing initial Gram stain, with the organism ultimately identified from an operative specimen. Identification of *A. aphrophilus* also prompted evaluation for potential oral and endovascular sources, revealing underlying dental disease without a clear acute odontogenic focus and no evidence of infective endocarditis. Taken together, these findings emphasize that negative initial microbiologic studies should not delay appropriate source control and further microbiologic investigation when the clinical and synovial fluid findings remain strongly suggestive of septic arthritis.

## Conclusions

*Aggregatibacter aphrophilus* should be considered a potential cause of septic arthritis even in patients who are afebrile, have a normal peripheral white blood cell count, and have negative initial Gram stain and cultures. When clinical findings remain concerning for septic arthritis, adequate operative sampling may be critical for identifying fastidious organisms. Isolation of *A. aphrophilus* should also prompt consideration of potential oral and endovascular sources. In this case, surgical source control followed by targeted ceftriaxone therapy resulted in complete clinical recovery.
